# Specific Measurement of Tethered Running Kinetics and its Relationship to Repeated Sprint Ability

**DOI:** 10.1515/hukin-2015-0127

**Published:** 2015-12-30

**Authors:** Filipe Sousa, Ivan dos Reis, Luiz Ribeiro, Luiz Martins, Claudio Gobatto

**Affiliations:** 1 School of Applied Sciences, University of Campinas, Jardim Santa Luiza, Limeira, São Paulo, Brazil; 2 Physical Education Faculty, University of Campinas, Barão Geraldo, Campinas, São Paulo, Brazil; 3 Department of Health Sciences, State University of Santa Cruz, Jorge Amado Road, km16, Salobrinho, Ilheus, Bahia, Brazil

**Keywords:** evaluation, force, velocity, power, impulse, work

## Abstract

Repeated sprint ability has been widely studied by researchers, however, analysis of the relationship between most kinetic variables and the effect of fatigue is still an ongoing process. To search for the best biomechanical parameter to evaluate repeated sprint ability, several kinetic variables were measured in a tethered field running test and compared regarding their sensitivity to fatigue and correlation with time trials in a free running condition. Nine male sprint runners (best average times: 100 m = 10.45 ± 0.07 s; 200 m = 21.36 ± 0.17 s; 400 m = 47.35 ± 1.09 s) completed two test sessions on a synthetic track. Each session consisted of six 35 m sprints interspersed by 10 s rest under tethered field running or free running conditions. Force, power, work, an impulse and a rate of force development were all directly measured using the sensors of a new tethered running apparatus, and a one-way ANOVA with Scheffé post-hoc test used to verify differences between sprints (p < 0.05). Pearson product-moment correlation measured the relationship between mechanical variables and free running performance. A total impulse, the rate of force development and maximum force did not show significant differences for most sprints. These three variables presented low to moderate correlations with free running performance (r between 0.01 and −0.35). Maximum and mean power presented the strongest correlations with free running performance (r = −0.71 and −0.76, respectively; p < 0.001), followed by mean force (r = −0.61; p < 0.001) and total work (r = −0.50; p < 0.001). It was concluded that under a severe work-to-rest ratio condition, power variables were better suited to evaluating repeated sprint ability than the other studied variables.

## Introduction

Strength-related variables and their role in functional performance have been the subject of several experimental and review papers (Cronin and Sleivert, 2005; Mirkov et al., 2004; Young, 2006). Maximum mechanical power is among the most commonly used biomechanical variables to predict performance (Cormie et al., 2011; Cronin and Sleivert, 2005). The use of maximum power to evaluate performance could be justified because power is composed of force and velocity – two important variables for general sports tasks (Cronin and Sleivert, 2005; Harris et al., 2007). However, it has been argued that other kinetic variables such as an impulse, work, or a rate of force development (RFD) could have even more influence than power in activities such as jumping or running (Cronin and Sleivert, 2005; Knudson, 2009; Mirkov et al., 2004). There is no consensus on the best variable to evaluate performance in running efforts, and differences in measurement techniques among the existing studies could be a reason behind the conflicting results.

Measurement of kinetic variables is often influenced by force application techniques and movement patterns (Morin et al., 2011). Specific protocols and ergometers have been designed to enhance evaluation specific to performance in a given sport. Some sport performances have thus been evaluated using a construct called “repeated sprint ability” (RSA). By definition, RSA is the ability to perform maximal sprints of short duration (≤10 s) repeatedly with brief (≤60 s) periods of recovery (Dawson, 2012; Girard et al., 2011). RSA is an important aspect of team sports since the significant reductions in sprint intensity towards the end of a match can be responsible for the final outcome of the game (Girard et al., 2011). In rugby, field hockey, soccer, and lacrosse, RSA is commonly evaluated by emulating the most intense work-to-rest ratio that is likely to occur in the chosen sport (Dawson, 2012).

Despite interesting analyses emerging from the study of RSA, it is commonly evaluated based only on the time taken to complete the efforts (Dawson, 2012). A time trial provides the coach with important information, but detailed sprint kinetics could help to guide training interventions. The relationship between power and the development of velocity in individual running sprints is often noted (Rabita et al., 2015; Rumpf et al., 2014). However, it is argued that the vector of force, the impulse or even the RFD should have more focus in the study and training of running performance, since the impulse-momentum relationship (Newton’s Second Law of Motion) is what links kinetics to movement kinematics (Knudson et al., 2009). This is a valid question when considering an individual sprint, and should also be taken into consideration in the study of repeated sprints. Unfortunately, there is a lack of research measuring kinetics directly in sprint running, which could arise from the difficulty in measuring such variables while running in the field. To overcome the difficulty of kinetic variable measurement, studies usually use cycle ergometers to infer kinetic capabilities (Bishop and Edge, 2006; McGawley and Bishop, 2006), but this approach lacks specificity when evaluating running-based sports. An alternative for measuring sprint kinetics with a similar movement pattern to field running is the tethered running model (Carling et al., 2012; Morin and Seve, 2011).

Tethered running is an evaluation model in which the runner performs sprints while they are tethered by their waists to a dynamometer, which measures the drag force exerted during the run (Carling et al., 2012; Lakomy, 1987). Usually, tethered running is performed at a laboratory using a non-motorized treadmill (Morin et al., 2011), but more recently, adaptations to field running sprints were presented (Lima et al., 2011). Tethered running variables have proved to be closer to free running performance than those measured using cycle ergometers (Lima et al., 2011). Several force and power variables could be measured using tethered running; however, the study of their influence on RSA is an ongoing process. Thus, using the tethered running model it would be valuable to evaluate which of the potential kinetic variables are more important to evaluate RSA performance, and suggest which variables should be focused upon in sports training.

This study investigated the kinetic variables that were likely to influence repeated sprint performance in field running. The hypothesis for this investigation was that power related variables would be better related to RSA than the others, since mechanical power is commonly associated with performance. The alternative hypothesis was that there were other kinetic variables better related to RSA. For that, several potential variables were compared regarding their sensibility to fatigue and a relationship with free running performance in RSA. Selection of kinetic variables better related to RSA could optimize sprint running analysis and training. Specifically, this analysis aimed to verify sensitivity to fatigue and correlation to repeated sprint time trials for force, power, the impulse, work, and the RFD. We performed this analysis using a protocol with an intense work-to-rest ratio, in order to compare the selected variables under different fatigue conditions for each given subject.

## Material and Methods

### Participants

A highly trained sample of sprint athletes was recruited. Participants were required to have a performance reaching at least 80% of the world record in at least one sprint modality for inclusion in the study. This criterion was set to ensure good sprint running kinematics of all participants. Thus, nine male sprint runners (age = 20.1 ± 1.9 years; body mass = 68.46 ± 6.18 kg, body height = 1.78 ± 0.05 m, body fat = 4.7 ± 1.18%) were recruited for this investigation. The mean best sprint performances of the subjects between 100, 200 and 400 m are displayed in Table 1. Despite the fact that team sport athletes would be more experienced in repeated sprint situations, track sprinters were recruited to take part in this investigation to ensure good running kinematics over the protocol. All participants were informed of the procedures and voluntarily gave written consent. Study procedures were approved by the institutional ethics committee for research involving humans of the São Paulo State University and complied with the ethics standards set in the Declaration of Helsinki.

### Procedures

On two occasions interspersed by 24–72 hours, participants performed a repeated sprint protocol in free or tethered running on the field. The repeated sprint protocol called the running-based anaerobic sprint test (RAST) (Zagatto et al., 2009) consisted of six 35 m sprints, interspaced by 10 s of passive rest. All testing took place on a synthetic track starting at the same time of the day (~9 am). Time trials were measured using a photocell arrangement under both conditions (Speed Test 6.0 standard®, CEFISE, Brazil). Each session began with a standardized warm-up consisting of 5 min running at moderate intensity with an additional minute at higher intensity (Wittekind et al., 2012). In both sessions, sprint runners used the same running shoes and lightweight clothes and were instructed to maintain the same food intake and hydration habits throughout the duration of the study. The sprint starting position was standardized as the participant standing with their preferred foot forward. All subjects had previous experience in using the prototype for at least 2 to 4 weeks, and were thus familiar with the tethered field running system.

### Tethered field running system

**Figure 1:**
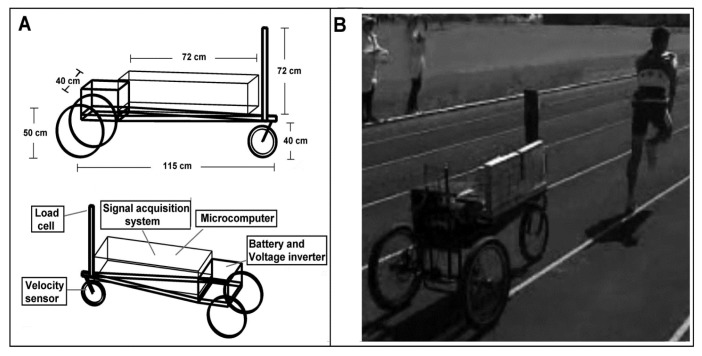
Specifications for the apparatus used during the tethered running condition (A) and example of application in a sprint (B).

An adaptation of a recently presented apparatus for tethered field running was used (Lima et al., 2011). It consisted of a rigid metal tricycle connected to the runner. Runners wore a nylon belt commonly used for load sprinting and were attached to the prototype by an inextensible steel cable (Figure 1). A load cell (CSL/ZL-250, MK Controle e Instrumentação Ltda, Brazil) placed on the high frontal pole of the prototype and attached to the cable was used to measure the athlete’s drag force. This load cell had its height adjusted according to the runner’s stature to maintain its horizontal orientation. A 2 m cable was used to smooth variations in its orientation resulting from ground contact (Lakomy, 1987). Four evenly spaced magnets placed in the front wheel of the tricycle were used to measure the horizontal displacement of the system. A Hall Effect sensor fixed at the wheel shaft captured a pulse every time one of the magnets passed it (i.e. every 31 cm of horizontal displacement). A disk brake in both rear wheels enabled the imposition of resistance, set at 9% of body weight. This load was chosen based on pilot investigations, undertaken to find the minimal load that prevented the load cell from losing its continuous signal during a sprint using the apparatus. Previous studies using sled towing had determined that resistances up to 12.6% BW offered minimal disruption in the running kinematics (Lockie et al., 2003; Maulder et al., 2008).

Signals from the load cell and Hall Effect sensors were recorded at 1000 Hz. The load cell signal was smoothed using a fourth-order Butterworth low-pass filter with cutoff frequency of 10 Hz. Conversion into force units (N) was performed by linear calibration using barbells attached to the load cell (acceleration of gravity considered as 9.81m·s^−2^). As stated, the Hall Effect sensor provided discrete information every 31 cm of displacement, despite the signal acquisition frequency. Displacement data was thus interpolated through a “spline” function to reach the force signal frequency of 1000 Hz.

**Figure 2:**
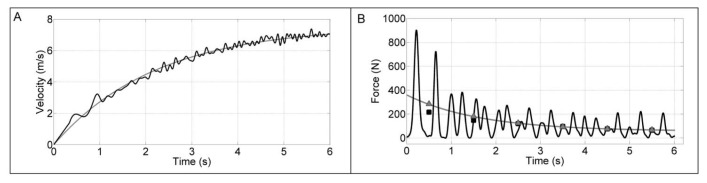
Example of signals captured by the prototype. Panel A shows velocity (A – black line) at 1000 Hz. Only for this example, velocity was modeled using V = Vmax *(1- e^−t/tau^) for smoothness (A – grey line). Panel B shows force signal at 1000 Hz obtained by the load cell (B – black line) and force obtained by the product between body mass and acceleration (B – grey line), as it can be seen in Morin and Seve (2011). Lastly, black squares and grey triangles in panel B represent the mean for each second for force data obtained by the load cell and calculated using acceleration, in order to exemplify its similarity in magnitude and behavior

Some aspects regarding the reliability and validity of this tethered field running system must be noted, and were investigated during pilot testing. Sprint time recorded by the photocell arrangement and by the prototype presented a difference of 1.3%. In addition, by logical validity, it was expected that the measured force would have an effect on the velocity development. For all sprints, force averaged per second presented significant correlations with gains in velocity at the corresponding time interval (Pearson product-moment between 0.96 and 0.99; *p* < 0.001). An example of velocity obtained by the prototype in a 35 m sprint is presented in Figure 2-A. Figure 2-B displays a comparison between force directly measured by the ergometer used in this study and estimated by acceleration for a given sprint. Test-retest analyses had been previously performed during pilot testing with another set of participants using intraclass correlation coefficients. Those for power, force and velocity (data pending publication) were significant between 0.70 and 0.82, attesting the reliability of the method.

### Description of the variables

Force, power, the impulse, work and the RFD were measured during the tethered field sprints. Velocity and time to complete each 35 m were also recorded. Force was directly obtained by the load cell signal. The velocity of the apparatus-athlete was calculated using the first derivative of displacement in time. Power was calculated as a direct product between the horizontal oriented force and velocity. In a similar way, the impulse was obtained as an integral of the force-time curve. Work was calculated as the sum of each product between the force and the respective displacement at each millisecond. The RFD was calculated as the first derivative of force in time. All variables were calculated based on the force and/or displacement component, which was horizontally-oriented.

Power, force and velocity were presented as maximal and mean. Maximal values were determined based on data averaged over 1 s intervals. Mean values were calculated as the data mean over the entire sprint. The RFD was presented as the highest value among each millisecond, while work and the impulse were presented as the total calculated for each given sprint. Except for velocity, all variables were normalized to body mass.

### Statistical analysis

Statistical analysis was performed using the MatLab statistical toolkit (version 4.0, Mathworks Inc., USA). Lilliefors and Levene’s tests were used to confirm the normal distribution and homoscedasticity of data. The results were displayed as mean ± SD. A one-way analysis of variance (ANOVA) for paired samples was used to identify significant changes along the six sprints for the time to complete the trial, velocity, force and power parameters, as well as the RFD, work and the impulse, all measured in the tethered running condition. When significant differences were found for one of those variables between the six sprints, a Scheffé *post-hoc* analysis was used to perform pairwise comparisons. The Scheffé analysis was chosen because of its property of coherence with the ANOVA results. Pearson’s product-moment correlations were evaluated between all the mechanical variables obtained in the tethered running condition and the performance in the free running condition. The correlations between variables in the tethered and free running conditions were measured respecting the sprint order of the repeated sprint protocol, to ensure a similar fatigue status. The guidelines set by Cohen were used to interpret the magnitude of the correlation coefficients, those in the order of 0.10 being “small”, those > 0.1 and ≥ 0.30 “medium” and those >0.30 and ≥ 0.50 “large” (Cohen, 1988; Hemphill, 2003). The level of significance for all statistical analyses was set at *p* < 0.05.

## Results

There was a significant increase in time to complete each sprint, and thus, the velocity parameters decreased (Table 2). Among the kinetic variables, the ANOVA and *post-hoc* analyses showed a total impulse, the RFD and maximum force as less sensitive to fatigue along the succession of sprints (Table 2). In turn, mean force, mean power and total work presented differences from the third sprint onwards, as did maximum power from the fourth sprint onwards. Maximum and mean values for power, mean force and total work were among the most sensitive variables for the fatigue effect.

**Figure 3:**
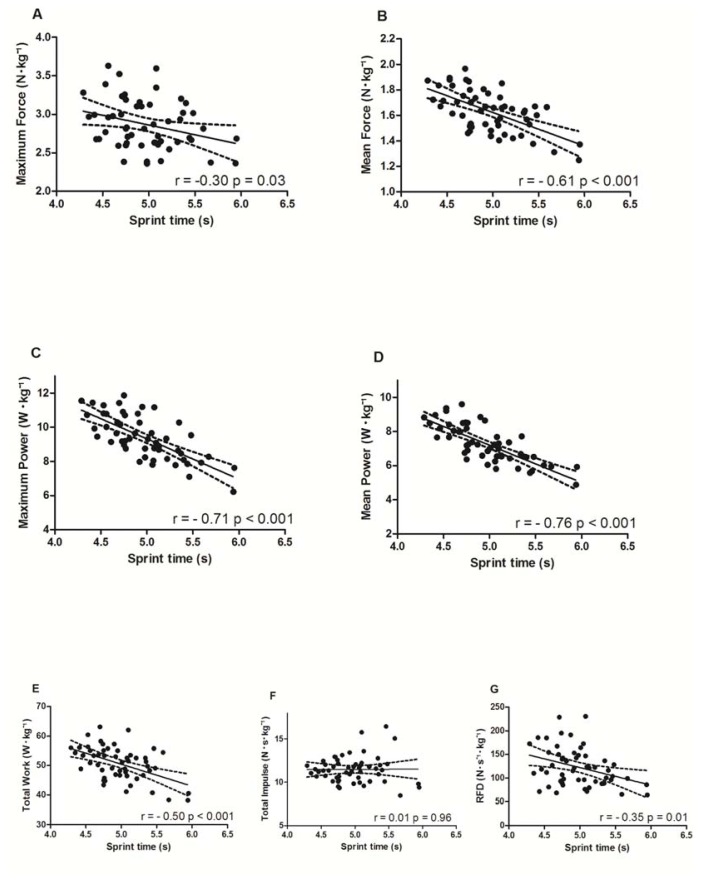
Relationships (solid line) and the 95% confidence interval (dashed line) between performance in free running and maximum force (A), mean force (B), maximum power (C), mean power (D), total work (E), a total impulse (F) and the RFD (G) measured in tethered field running. Data of each sprint were compared between conditions following sprint order in RAST to preserve a similar fatigue status

In addition to their low sensitivity to fatigue, a total impulse, the RFD and maximum force also demonstrated small relationships with the time to complete the sprint performed in the free running condition (Figure 3). Medium to large significant relationships were found between the tethered running variables and free running performance for maximum and mean velocity (−0.61 and −0.60; *p* < 0.001), maximum and mean power, mean force, and total work (Figure 3).

**Figure 4:**
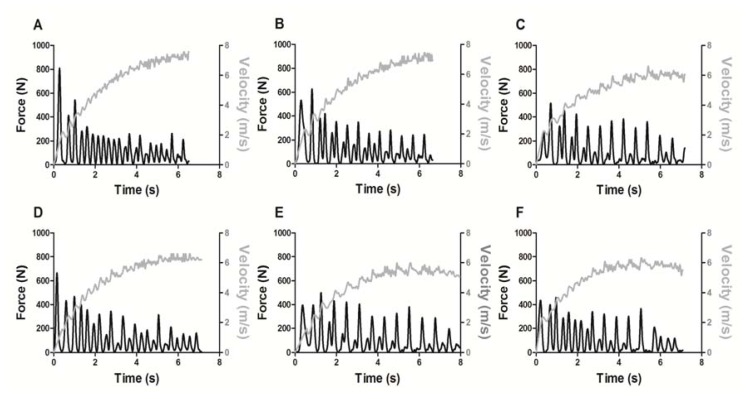
Typical example of the raw data of the force (black lines) and the velocity (grey lines) performed during the six sprints in the tethered field condition. The order of the sprints is following alphabetical order

Figure 4 displays a graphical example of force and velocity developed in each of the six sprints during the RSA protocol. It is possible to see a decrement in the force signal over the six sprints, along with a decrease in velocity, suggesting the presence of fatigue in the protocol.

## Discussion

Although there is existing data relating running sprint performance to the impulse, work, the RFD, power and force (Harris et al., 2008; Hunter et al., 2005; Morin et al., 2011), this study is the first to measure all of these variables in the same field sprint and while running in a repeated sprint situation; previous investigations had used squat jump machines (Harris et al., 2008), treadmills (Carling et al., 2012), or measurements during only one or a few steps (Hunter et al., 2005). Here, the kinetic variables were all directly compared regarding their sensitivity to fatigue and their relationship to sprint running performance. A relatively non-specific method of measurement, such as using countermovement jumps, squats and throws, has been suggested as the reason for weak correlations between the obtained kinetic variables and performance in running (Harris et al., 2008). A major advantage of the current study is the opportunity to measure all kinetic variables directly during an entire sprint run. In the studied conditions, power variables, total work and mean force were identified as more sensitive to fatigue and more related to free running performance.

Studies using laboratory tethered running present similar results for power to those of the data presented here (Table 2). Values ranging from 11.1 to 22.4 W·kg^−1^ can be found for power in individual sprints lasting from 8 to 10 s (Chia and Lim, 2008; Morin et al., 2011). Higher power values were found using a tethered field system similar to ours (Lima et al., 2011), but that was credited to the twofold imposed resistance. Previous investigations of the relationship between imposed resistance and power in running supports the idea that power should be greater in the resistance used in Lima et al. (2011) (18% BM or ~ 120,07 N) than that used here (9% BM or ~ 60,44 N). In contrast, similar results found for power measured in previous studies using laboratory tethered running imply a similarity in the resistance of previous treadmills and the one imposed in our field model. There is no consensus as to the optimum resistance to enhance power results, and it may vary according to the movement pattern, thus there is evident need for further investigation (Cormie et al., 2011; Cronin and Sleivert, 2005). However, it should be noted that under a similar resistance condition, the results presented in the current investigation are in line with data from previous studies.

Other studies showed lower values for the impulse than those presented in this investigation (Table 2) (Hunter et al., 2005; Kawamori et al., 2014; Slawinski et al., 2010). Regarding the differences between the impulse and work presented in the current investigation and in other studies, the tethered field running system enabled continuous measurement of the horizontal pulling force for an entire sprint. The continuous measurement of force allowed for the calculation of the total impulse and total work performed during the whole sprint, rather than only considering the ground contact of a few footsteps (Hunter et al., 2005; Rabita et al., 2015). In this way, these results are a new approach to studying the impulse and the work performed while running. Instead of using data from a single step, or from a few steps, the total amount of the impulse and work can be obtained using the methodology from the current study. The athlete’s capacity for continuously exerting the impulse and performing work can thus be measured using the total impulse and total work. It is plausible to think that the total amount of the impulse and work exerted is a valuable parameter for measurement in the study of fatigue effect over successive short sprints. The importance of these variables in the study of fatigue is illustrated in the results from the current study.

Differences in time to complete the sprints demonstrated the fatigue effect according to the protocol used in this research. Based on the number of significant differences presented in the *post-hoc* analyses (Table 2), the fatigue had a lower effect on maximum force and the RFD, suggesting the sprinter was able to maintain his maximum amount of force exertion in the first five sprints. However, fatigue affected overall force exertion during the 35 m sprint, as can be seen by the significant lower mean force from the third sprint onwards. A graphical example supporting this assumption is provided in Figure 4, where the decrement in force becomes visible along the sprints, while the sprint time is increasing. Consequently, based on the significant differences presented by the ANOVA and *post-hoc* analyses, as fatigue increased the time to complete a given sprint, it also decreased the ability to exert force (Delextrat et al., 2013; Girard et al., 2011). In this way, the total impulse performed during a sprint was not significantly different along the protocol. Calculation of the total impulse performed in the current study demonstrated an unusual result: the compensation of the increase in sprint time by lower application of force, resulting in an unaltered total impulse. This result would not be visible using the calculation of the impulse in a single foot step. Authors aiming to investigate the fatigue effect in multiple bouts of repeated sprints, or in sports matches, should consider applying the method for the total impulse calculation presented here, in order to further investigate relationships between force and the impulse during RSA actions.

In addition to investigation of the fatigue effect in the repeated sprint protocol, another crucial aspect of the selection of useful parameters to measure the RSA is their interaction with performance. The relationship between the measured kinetic variables and performance in free running was investigated in the current study. Overall, maximum and mean power were better correlated with performance than total work and the total impulse, findings which are contradictory to the results obtained in previous studies (Harris et al., 2008; Hunter et al., 2005). Considering previous investigations, the poor correlations between performance and the impulse from the current research may seem unexpected. However, as discussed, the method used for calculating the impulse in this study was different to that of previous research (Hunter et al., 2005; Kawamori et al., 2014; Slawinski et al., 2010), which would have affected the degree of significant relationships with sprint performance. In this research, the impulse results considered all foot strikes in the sprint, potentially making it more valid for the analysis of running performance.

The results presented here regarding the small and medium correlations found between sprint running performance, the RFD and maximum force (Figures 3-A and 3-G) are in accordance with previous literature (Mirkov et al., 2004). Based on these collective results, it is possible that the rate and magnitude of the initial force exertion is not as important for performance in sprints as long as 35 m. In contrast, strong relationships between sprint running performance and peak power, mean power, and mean pulling force were presented in the current study (Figures 3-B, 3-C and 3-D), as well as in previous investigations (Morin et al., 2011). This supports the importance of these variables to performance for extended sprints over 30 m or above.

There has been recent discussion about the importance of mechanical power in sports performance (Cronin and Sleivert, 2005; Knudson, 2009). Usually, inconsistent results among studies investigating the relationship between mechanical power and sports performance arise from an unclear definition of the term ‘power’, a lack of specificity regarding the measures and methods used to calculate power, and in the times in which this power occurs (Knudson, 2009). In fact, studies investigating the effect of fatigue over successive sprints and in sports matches, or the relationships between kinetic variables and sprint performance, are usually based on data collected using exercises such as elbow extension, squat machines, heights of squat and countermovement jumps, and torque in isometric contractions (Harris et al., 2008; Mirkov et al., 2004; Nedelec et al., 2014). The current investigation measured several kinetic variables, including power, during the specific movement pattern of sprint running, with details of how and where the sensors were placed, in attempt to assure the interpretation and the potential for comparison by future studies. Mechanical definitions were used to calculate all kinetic parameters included in the investigation, avoiding the use of concepts of athletic characteristics or neuromuscular elements. In this way, the strong correlations between power and running performance contribute to the discussion about which variables are better correlated to performance in a repeated sprint protocol. If measured specifically, mechanical power, the total work exerted, and the mean force are better related to sprint running performance than maximum force and the RFD (Figure 3), suggesting greater importance of the former to RSA events. Rather than abrupt force exertion (high rate and amount), to have good RSA performance force must be continuously exerted in high amounts, resulting in higher mean force, work and even power. Furthermore, power variables also take into consideration the linear velocity of the runner, which is expected to be important to RSA.

Based on the results presented in this research, coaches and athletes aiming to improve RSA should embrace training techniques that enhance force production, peak power and mean power. For this reason, the exercise program should focus on movement specificity. Hypertrophy and general power exercises can improve running performance, but the optimum transference of the strength gains requires specific training (Kawamori et al., 2014; Young, 2006; Zafeiridis et al., 2005). Furthermore, while training to develop maximal force requires high tension of non-fatigued muscles (Harris et al., 2007), power enhancement would need fast movements (Cormie et al., 2011) that could not be possible using high loads. A good alternative for improving specific power in running is sprint training pulling weighed sleds (Young, 2006), which together with a signal acquisition system such as the one used in the current study would provide feedback of the developed force and power.

Calculations of decrement scores commonly seen in repeated sprint protocols were not performed in the current investigation. The extensive work of Glaister et al. (2008) clearly shows that irrespective of the calculation method, fatigue indexes could present very poor re-test reliability (variation = ~25–45%). Conclusions based on such parameters could therefore be affected by a high variation embedded in its calculation. In the current investigation, ANOVA and *post-hoc* analyses were able to indicate the variables that were more affected by fatigue, enabling us to draw the presented conclusions without using fatigue index calculations. Although these calculations do have value, the current investigation was able to notice a lack of sensibility to fatigue for maximum force, a total impulse and the RFD, excluding them as potential variables to measure performance in RSA.

Among the limitations of this study there is the innovative prototype used to measure all kinetic variables, since it had not been used in previous investigations. However, considering the validation aspects presented in the methods section, together with the possibility of measuring kinetic variables in field running sprints, such a limitation may be seen as an advantage. The ergometer used resembles a typical weighed sled commonly used in sprint training. Another limitation is a relatively low (n = 9) number of participants. Even so, the chosen participants were all high level athletes, and to perform experimental measurements on this type of individuals can be difficult. Lastly, team sport athletes would have better performance in the repeated sprints protocol, however, we chose to use elite sprinters to ensure good running kinematics. The results presented here could be different if team sport athletes were used instead of track runners.

Based on the presented results, it can be concluded that a total impulse, the RFD and maximum force do not seem to be the best variables through which to detect fatigue among successive sprints. The other kinetic variables measured in the study (i.e. power, mean force, and total work) were more sensitive to fatigue over the proposed protocol. The total impulse, the RFD, and maximum force presented only small to medium correlations to performance. It can be concluded that power related variables were better related to overall performance in the RSA protocol among the studied variables, confirming this study hypothesis. Athletic training and testing should consider inclusion of power drills using methods as specific as possible to enhance and/or evaluate repeated sprint running performance.

## Figures and Tables

**Table 1:** Descriptive data (MD ± SD) characterizing the performance level of the sprinters in their best scores between 100 m, 200 m and 400 m

	Time (s)	WR (%)
100 m (n = 2)	10.45 ± 0.07	90.9 ± 0.7
200 m (n = 3)	21.36 ± 0.17	87.8 ±1.6
400 m (n = 4)	47.35 ± 1.09	90.3 ± 1.5

Time – Best time trial score recorded in the last official competition before procedures. WR – Best time trial score relative to the world record in the same year of the last official competition before procedures.

**Table 2:** Descriptive data (MD ± SD) for mechanical variables in the 6 sprints on the tethered running condition, with ANOVA and post-hoc comparison between them

	Sprint 1	Sprint 2	Sprint 3	Sprint 4	Sprint 5	Sprint 6	ANOVA
Time (s)	6.42 ± 0.20	6.44 ± 0.23	6.97 ± 0.52	7.09 ± 0.60^a^,^b^	7.87 ± 0.83^a^,^b^,^c^,^d^	7.50 ± 0.69^a^,^b^	(<0.001)
V_max_ (m·s^−1^)	7.16 ± 0.20	7.17 ± 0.38	6.36 ± 0.46^a^,^b^	6.23 ± 0.59^a^,^b^	5.42 ± 0.59^a^,^b^,^c^,^d^	5.68 ± 0.58^a^,^b^,^c^,^d^	(<0.001)
V_mean_ (m·s^−1^)	5.45 ± 0.16	5.44 ± 0.20	5.03 ± 0.35^a^,^b^	4.95 ± 0.37^a^,^b^	4.47 ± 0.41^a^,^b^,^c^,^d^	4.68 ± 0.40^a^,^b^,^c^	(<0.001)
F_max_ (N·kg^−1^)	2.87 ± 0.25	3.09 ± 0.31	2.92 ± 0.98	2.79 ± 0.30	2.80 ± 0.41	2.74 ± 0.22 b	(0.01)
F_mean_ (N·kg^−1^)	1.75 ± 0.13	1.77 ± 0.14	1.63 ± 0.13^a^,^b^	1.60 ± 0.16^a^,^b^	1.52 ± 0.15^a^,^b^,^c^	1.51 ± 0.11^a^,^b^,^c^	(<0.001)
P_max_ (W·kg^−1^)	10.46 ± 0.89	10.41 ± 0.94	9.90 ± 0.95	9.22 ± 0.93^a^,^b^	8.03 ± 0.95^a^,^b^,^c^,^d^	8.32 ± 0.59^a^,^b^,^c^,^d^	(<0.001)
P_mean_ (W·kg^−1^)	8.44 ± 0.61	8.46 ± 0.76	7.30 ± 0.62^a^,^b^	6.96 ± 0.55^a^,^b^	6.07 ± 0.66^a^,^b^,^c^,^d^	6.33 ± 0.35^a^,^b^,^c^,^d^	(<0.001)
Work (J·kg^−1^)	54.2 ± 4.6	54.5 ± 5.3	50.8 ± 4.3^a^,^b^	49.5 ± 6.6^a^,^b^	47.6 ± 5.6^a^,^b^,^c^	47.5 ± 4.7^a^,^b^,^c^	(<0.001)
Impulse (N·s·kg^−1^)	11.27 ± 1.09	11.43 ± 1.07	11.47 ± 1.33	11.41± 2.00	11.98 ± 2.01	11.41 ± 1.82	(0.48)
RFD (N·s^−1^·kg^−1^)	140 ± 50	134 ± 41	119 ± 34	128 ± 37	122 ± 47	96 ± 28 a	(0.01)

Post-hoc analysis:

a p < 0.05 in relation to Sprint 1;

b p < 0.05 in relation to Sprint 2;

c p < 0.05 in relation to Sprint 3;

d p < 0.05 in relation to Sprint 4.

V_max_ – Maximum velocity; V_mean_ – Mean velocity; F_max_ – Maximum force; F_mean_ – Mean force; P_max_ – Maximum power; P_mean_ – Mean power
